# Fracture load and failure patterns of zirconia and 3D-printed resin endocrowns: an *in vitro* study

**DOI:** 10.7717/peerj.21602

**Published:** 2026-08-31

**Authors:** Okba Mahmoud, Muaiyed Mahmoud Buzayan, Selva Malar Munusamy, Aeman Elkezza, Eshamsul Sulaiman, Maysara Adnan Ibrahim

**Affiliations:** 1 College of Dentistry, Department of Clinical Sciences, Ajman University, Ajman, United Arab Emirates; 2 Department of Restorative Dentistry, Faculty of Dentistry, Universiti Malaya, Kuala Lumpur, Malaysia; 3 Department of Restorative Dentistry, College of Dental Medicine University of Sharjah, Sharjah, United Arab Emirates

**Keywords:** Endocrown, Zirconia, VarseoSmile Crown Plus, Fracture resistance, 3D-printing

## Abstract

**Background:**

Endodontically treated teeth are more prone to fracture and often require durable restorative solutions. Endocrowns offer a conservative alternative to conventional post-and-core systems. This *in vitro* study compared the fracture load and failure behavior of milled zirconia and 3D-printed resin endocrowns.

**Materials and methods:**

Eighteen extracted human molars were endodontically treated and standardized, then prepared for endocrown restorations using a uniform design (two mm occlusal reduction with a four mm intracoronal extension). Specimens were randomly assigned to receive either Computer-Aided Design (CAD)/ Computer-Aided Manufacturing (CAM) milled zirconia (ZR) or 3D-printed resin-based endocrowns fabricated from VarseoSmile Crown Plus (VS) (*n* = 9 per group). Preparations were digitally captured with an intraoral scanner, restorations were designed using CAD software and fabricated by the respective workflows, and all units were cemented with dual cure self-adhesive resin cement. After controlled storage, specimens underwent compressive loading to failure; maximum fracture loads were recorded, and failure modes were categorized according to whether damage was confined to the restoration or involved the tooth structure. Data were assessed for normality and analyzed using an independent samples *t*-test with Welch correction at a significance level of *p* < 0.05.

**Results:**

Statistical analysis revealed a highly significant difference in fracture load between the two groups (*p* < 0.001), with ZR demonstrating higher strength (3,615.36 ± 792.74 N) compared with VS (1,395.09 ± 149.24 N). However, tooth structure involvement occurred in 8/9 ZR specimens (88.9%), of these, five specimens (55.6% of the total group) were classified as complex/destructive failure, whereas VS failures were simple and confined to the restoration in 5/9 specimens (55.6%), indicating a less severe and potentially repairable failure pattern.

**Conclusion:**

The ZR group showed substantially higher fracture load, but failures more frequently involved tooth structure. VS group exhibited lower strength, yet a higher proportion of simple, potentially repairable fractures confined to the restoration, highlighting a tradeoff between maximum load capacity and repairability. Within the limitations of this *in vitro* study, these findings support the use of 3D-printed resin endocrowns in selected clinical situations where a tooth preserving endocrown design and digital workflow efficiency are prioritized, and where less severe failure behavior is desirable.

## Introduction

Endodontically treated teeth often exhibit substantial structural loss, leaving them susceptible to fracture under functional loading (Plotino et al., 2017). Endocrown restorations have gained popularity as an alternative to post-and-core systems or full-coverage crowns because they preserve tooth structure, enhance retention, and demonstrate favorable clinical outcomes (Belleflamme et al., 2017; Bindl & Mörmann, 2001). Endocrowns are designed as single-unit restorations that combine the crown and core, making them well suited for molars and premolars (Pissis, 1995). Their extension into the pulp chamber or root canal orifices provides a coronal seal and mechanical retention without the need for a separate post (Dietschi et al., 2008). The longevity of endocrowns is largely influenced by the mechanical performance of the material, including fracture load, wear characteristics, and bonding capacity (Dartora et al., 2021).

Endocrowns have been fabricated from several material classes, most commonly glass-ceramics such as lithium disilicate, zirconia-based ceramics, resin-based Computer-Aided Design (CAD)/ Computer-Aided Manufacturing (CAM) materials and hybrid/composite materials, each offering distinct clinical advantages. Lithium disilicate is often highlighted for its bonding potential and aesthetics, while zirconia remains a valuable option because of its high strength, toughness, and suitability for situations where greater mechanical reliability is desired (Zhang & Kelly, 2017). More recently, resin-matrix ceramics and composite-based materials have also been introduced as alternative options with different stress-distribution and repairability profiles (AlDabeeb et al., 2023).

Numerous investigations have evaluated the fracture load and bonding behavior of zirconia restorations (Raigrodski et al., 2012; Sailer et al., 2007). However, ongoing advances in dental materials highlight the importance of assessing newer options (Al-Ramadan et al., 2024; BEGO, 2025; Duret, Blouin & Duret, 1988; Lin et al., 2012; Tribst et al., 2019; Zarone et al., 2019). Among these, the 3D printed resin tested in this study, a ceramic-filled 3D-printed hybrid resin, has emerged as a viable material for definitive restorations. Previous studies have reported marginal and internal fit values comparable with milled resin-matrix ceramic crowns, such as Vita Enamic, as well as flexural strength values within clinically acceptable limits (Al-Ramadan et al., 2024; BEGO, 2025). Furthermore, 3D-printing supports efficient workflows and customized design (Sedrez-Porto et al., 2016; Tokar et al., 2024). However, its mechanical behavior relative to zirconia, specifically for endocrowns, remains insufficiently explored.

Because endocrowns function under considerable occlusal forces, their clinical performance depends greatly on the mechanical properties of the restorative material. These properties influence not only the magnitude of load the restoration can withstand, but also whether failure remains limited to the restoration or extends to the underlying tooth structure (Biacchi & Basting, 2012; Elsaka & Elnaghy, 2016).

In this context, endocrown materials may be fabricated through either subtractive or additive workflows. CAD/CAM milling represents the subtractive approach, whereas 3D-printing represents the additive approach, offering greater design flexibility and workflow efficiency (Al-Ramadan et al., 2024; Duret, Blouin & Duret, 1988; Tokar et al., 2024).

Zirconia-based ceramics, particularly 3Y-TZP, are recognized for their high flexural strength, which is commonly reported to range from approximately 900 to 1,300 MPa in contemporary reviews, making them suitable for high-load bearing posterior applications. More recently, multilayer zirconia systems that combine 3Y-TZP with higher-yttria zirconia, such as 5Y-TZP, have been introduced to improve translucency while maintaining a favorable balance of mechanical performance and aesthetic properties. By comparison, definitive 3D-printed resin materials generally demonstrate markedly lower strength values; published data for the 3D printed resin tested in this study have shown mean flexural strength values of 109.9 ± 15.8 MPa before ageing and 94.2 ± 11.7 MPa after ageing, consistent with manufacturer-reported values of 116 to 150 MPa. These quantitative differences highlight the need to determine whether newer printed resin materials can provide acceptable fracture behavior despite their lower intrinsic strength (Di Fiore et al., 2024; Ham et al., 2025).

Despite zirconia’s well documented performance, evidence directly comparing it with 3D-printed endocrown materials remains limited. As these materials are relatively recent, several aspects of their behavior have not yet been thoroughly investigated. It therefore remains unclear whether they can provide predictable mechanical performance while preserving the conservative preparation approach that makes endocrowns clinically attractive. This investigation therefore evaluates and compares the fracture load and failure patterns of multilayer zirconia and the tested 3D-printed resin endocrowns under in-vitro conditions. The null hypothesis states that no significant difference exists between the two materials in terms of fracture load or failure pattern.

## Materials and Methods

### Ethical approval

This study was approved by the Research Ethics Committee at Ajman University, UAE (Reference number: D-H-S-2023-DEC-6-20, Approval Date: 19 December 2023). This was an *in vitro* study performed on previously collected extracted human teeth. No human participants were recruited, and no patient-identifiable information was accessed. Therefore, informed consent was not required for the use of the extracted teeth in this laboratory-based investigation. The study adhered to institutional ethical standards and the principles of the Declaration of Helsinki.

### Sample size calculation

The sample size was calculated using the G Power software (version 3.1.9.4, Heinrich Heine University Düsseldorf, Germany). The input parameters were retrieved from the results reported by El-Damanhoury, Haj-Ali & Platt (2015). Based on an alpha level of 0.05, a statistical power of 0.90, and an effect size f of 0.88, the required total sample size was determined to be 16, evenly distributed over two groups (*n* = 8 per group). To account for potential specimen loss during specimen preparation and fracture testing, the final sample size was increased to 18, with nine specimens allocated to each group (*n* = 9).

### Tooth selection, eligibility, and storage

A total of 18 extracted human mandibular first molar teeth, free of caries, cracks, restorations, root resorption or previous endodontic treatment, were collected from patients undergoing orthodontic treatment or other dental extraction procedures. Teeth of similar size, shape and root morphology were selected to ensure sample homogeneity, with crown dimensions kept within a narrow range of approximately seven to eight mm in crown height, 10 to 12 mm mesiodistally, and 10 to 11 mm buccolingually; teeth with obvious anatomical variation were excluded. The teeth were stratified by size into small, medium, and large categories, and then distributed evenly between the two groups with random allocation within each category. They were stored in 0.1% thymol at room temperature until use. Eligibility was confirmed by preoperative periapical radiographs and microscopic inspection after ultrasonic root surface cleaning.

### Specimen preparation

Patency was verified with a size 10 K-file under magnification, and root canal preparation was performed using a WaveOne Gold Primary file with an X Smart motor (Dentsply Sirona, Ballaigues, Switzerland). After drying, the canals were obturated with BioRoot Flow, a calcium silicate-based bioceramic sealer (Septodont, Saint Maur des Fossés, France), in combination with matched WaveOne Gold gutta-percha cones (Dentsply Sirona, Johnson City, TN, USA) placed to full working length using a single cone technique. Periapical radiographs were taken to confirm a uniform, void free obturation. After obturation, the pulp chamber floor was sealed with a flowable composite resin (Filtek Z350 XT Flowable, 3M ESPE) before storage, and the specimens were then stored at 37 °C and 100% humidity for 7 days to allow complete setting of the sealer.

Following endodontic treatment, each tooth was mounted in an epoxy resin block (Mirapox 950-230 A and B; Miracon (M) Sdn Bhd, Malaysia) measuring 25 × 25 × 25 mm to ensure stability and procedural reproducibility. The specimens were allocated to two groups (*n* = 9 per group) for restoration with either zirconia or VarseoSmile Crown Plus endocrowns. The restorative, luting, and auxiliary materials used in the study are summarized in Table 1.

**Table 1:** Materials used in the study.

**Material class/type**	**Trade name**	**Manufacturer**	**Lot/Batch no.**	**Composition/source**
Multilayer yttria- stabilised zirconia	Nacera Pearl Natural	DOCERAM Medical Ceramics GmbH, Germany	5199190	Zirconium dioxide stabilised with yttrium oxide; 3Y-TZP and 5Y-TZP multilayer structure; hafnium oxide, aluminium oxide, and other trace oxides as minor components (Dental Direkt, 2024).
Ceramic-filled methacrylate- based 3D-printed resin	VarseoSmile Crown Plus	BEGO GmbH & Co. KG, Bremen, Germany	601195	4.4′-isopropylidiphenol, ethoxylated and 2-methylprop-2enoic acid, 30%–50% by mass inorganic filler (particle size 0.7 µm), silanised dental glass, methyl benzoyl formate, diphenyl (2,4,6-trimethylbenzoyl) phosphine oxide (Almualm et al., 2026).
Dual-cure self-adhesive resin cement	RelyX U200 Automix	3M ESPE, Seefeld, Germany	11262961	Base paste: glass powde treated with silane, 2-propenoic acid, 2-methyl 1,10- (1-[hydroxymetil]-1,2-ethanodlyl) ester dimethacrylate, triethylene glycol dimethacrylate (TEGDMA), silica treated silane, glass fiber, sodium persulfate and per-3,5,5-trimethyl hexanoate t-butyl; catalyst paste: glass powder treated with silane, substitute dimethacrylate, silica-treated silane, sodium ptoluenesulfonate, 1-benzyl-5- phenyl-acid barium, calcium, 1,12-dodecane dimethacrylate, calcium hydroxide, and titanium dioxide (Hammal et al., 2021).
Flowable nanocomposite resin	Filtek Z350 XT Flowable	3M ESPE, St Paul, MN, USA	10639739	Inorganic content fraction: Zirconia/ silica cluster filler and 20 nm silica filler 65 wt% Organic matrix: bis-GMA, TEGDMA, and bis-EMA (De Jesus et al., 2020).

**Figure 1:**
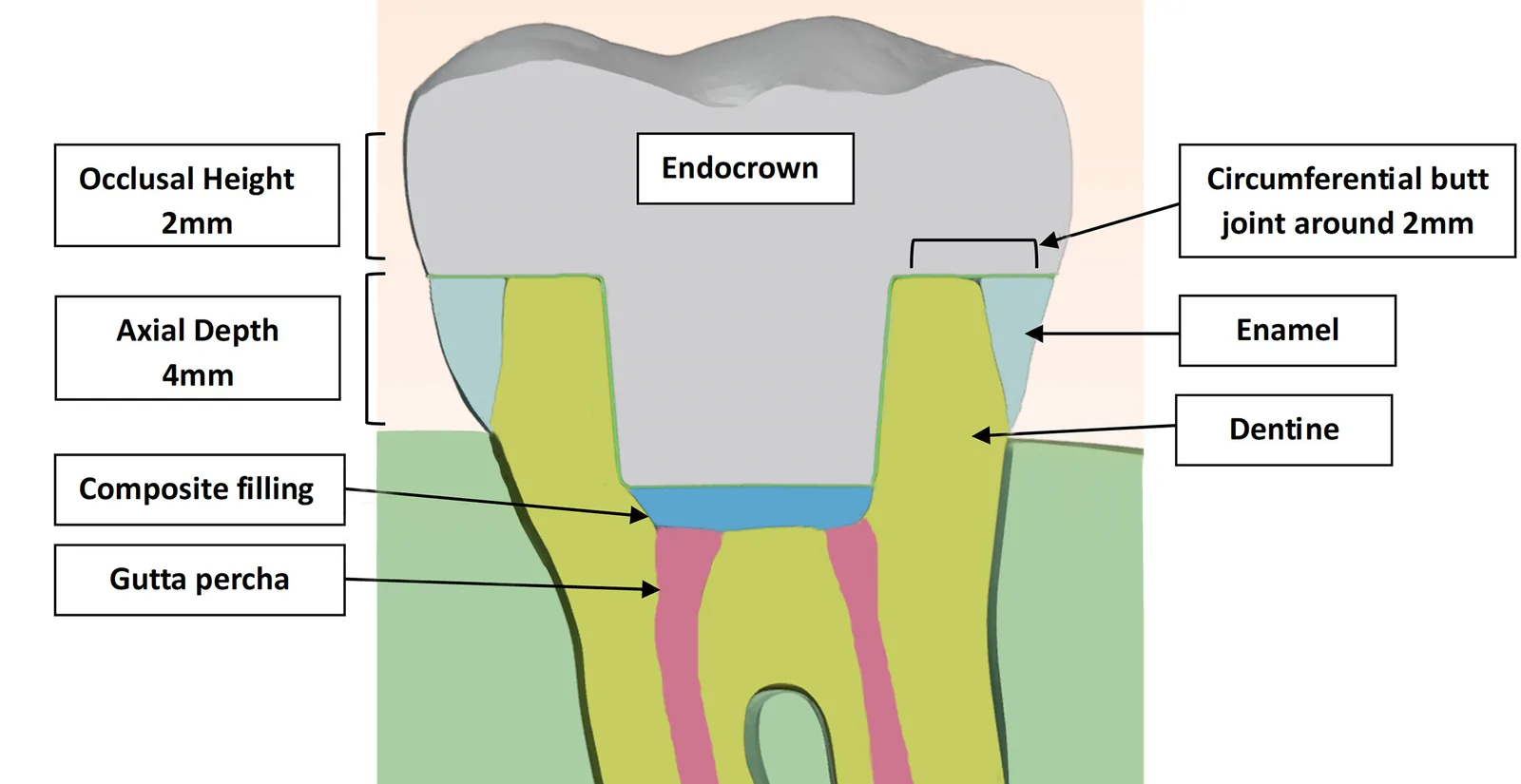
Schematic illustrating the preparation of the endocrown, showing the outline and the different layers involved.

**Figure 2:**
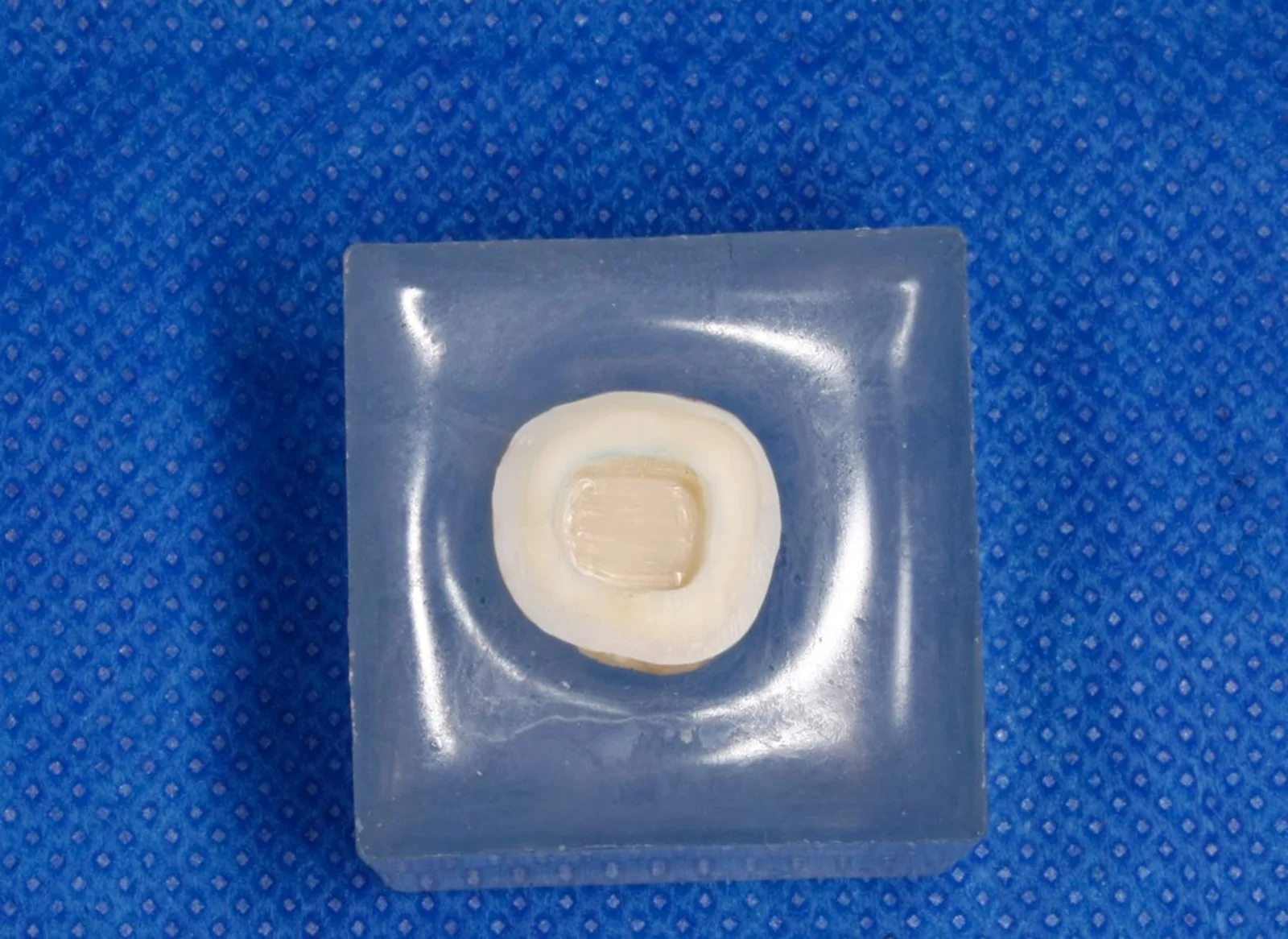
Occlusal view of the endocrown. Occlusal view demonstrating the butt joint preparation of the natural tooth prior to endocrown cementation.

Tooth preparation for endocrown restorations was performed manually by a single experienced specialist using a high-speed handpiece and diamond burs under water cooling, as this approach was considered more clinically relevant. Initial occlusal reduction was carried out using a straight round-end diamond bur (FG, SR 12, ISO 141/014), flattening of the occlusal surface and butt-joint refinement were performed with a wheel diamond bur (909G-031-FG), pulp chamber preparation and axial wall formation were completed using a tapered shoulder flat top round edge diamond bur (D.845KR.016.FG), and final refinement was carried out with a round end tapered diamond bur (D.850.016.FG). A uniform occlusal reduction of two mm and a central pulp chamber depth of four mm were prepared. To ensure standardization, the preparations were digitally verified after scanning using Blender (Blender Foundation, Amsterdam, The Netherlands) by measuring the axial wall taper at four points (mid buccal, mid lingual, mid mesial, and mid distal), and all specimens remained within the predetermined taper range of 8° to 10°. All sharp angles were rounded to minimize stress concentration (Figs. 1 and 2).

The endocrowns were fabricated using a digital workflow. Each prepared tooth was scanned individually with an intraoral scanner (TRIOS 4; 3Shape, Denmark) to obtain a specimen-specific digital preparation and fitting surface. The scans were then imported into CAD software (Blender for Dental v3.2; Blender Foundation, Australia), where the restorations were designed with a cement space of 50 µm. To standardize the external morphology, the same anatomical reference tooth was used to guide the occlusal design for all restorations. The reference tooth was obtained from the digital tooth library within the CAD software and was applied consistently to all specimens. This reference morphology was scaled and adjusted according to the mesiodistal and buccolingual dimensions of each specimen, while maintaining a comparable occlusal anatomy and an occlusal height of approximately two mm across samples.

Once the design was finalized, the digital files were transferred to a CAM system, where the endocrowns were milled using an inLab MC X5 dental milling machine (Dentsply Sirona) from Nacera Pearl Natural zirconia blocks (DOCERAM Medical Ceramics GmbH, Germany), a multilayer hybrid zirconia composed of 5Y-TZP and 3Y-TZP. This multilayer material combines mechanical strength and translucency for restorative applications. Following milling, the zirconia endocrowns were sintered according to the manufacturer’s recommended standard protocol for Nacera Pearl Natural, with a peak temperature of 1,500 °C and a total cycle time of approximately 7.8 h. For the tested 3D-printed resin group, the restorations were 3D-printed using a SOL 3D-printer (Ackuretta Technologies, Taiwan). Printing was performed with a layer thickness of 70 µm at a 0° build orientation. After printing, the restorations were cleaned using a CLEANI washing unit (Ackuretta Technologies, Taipei, Taiwan) with 96% ethanol, following the material manufacturer’s instructions. The restorations were then post-cured using a CURIE Plus unit (Ackuretta Technologies, Taipei, Taiwan) for 2 × 2 min, with the restorations inverted between curing cycles. No airborne-particle abrasion, etching, or separate primer was performed for the 3D-printed resin restorations before cementation.

The endocrowns were cemented to their respective teeth using 3M™ RelyX™ U200 Automix Self-Adhesive Resin Cement (3M ESPE, Seefeld, Germany) following the manufacturer’s guidelines.

For the zirconia endocrowns, the internal surfaces were airborne-particle abraded with 50 µm aluminum oxide particles at 2 bar pressure from a distance of 10 mm for 10 s to enhance mechanical retention and bond strength. After airborne-particle abrasion, the zirconia restorations were ultrasonically cleaned in distilled water and air-dried. No separate zirconia primer or tooth adhesive was applied, as the restorations were cemented using a self-adhesive resin cement according to the manufacturer’s protocol.

For both groups the cement was then dispensed directly into the internal surfaces of the endocrowns using the automix syringe, and each restoration was initially seated onto the prepared tooth with firm finger pressure to ensure complete seating and adaptation. To standardize seating and help control cement thickness, a static load of five kg was applied during cementation and maintained until completion of light curing. Excess cement was carefully removed from the margins using a microbrush and dental explorer. The cement was then light cured for 20 s per surface (buccal, lingual, mesial, and distal) to ensure thorough polymerization. After the completion of the cementation process, the samples were stored for 24 h in distilled water at 37 °C to simulate the oral environment and allow the resin cement to fully set.

**Figure 3:**
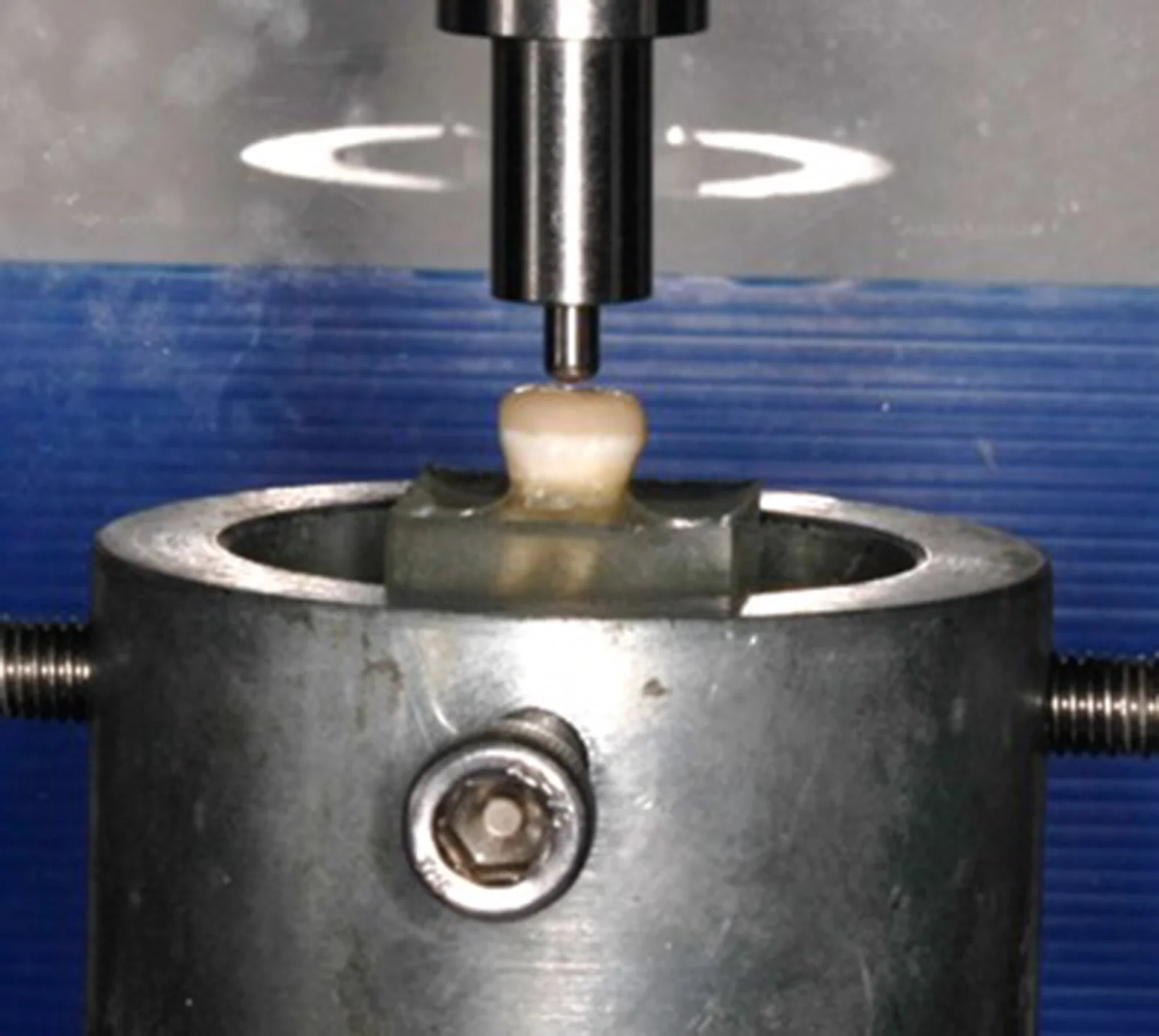
Sample fixed in the universal testing machine (UTM) for the fracture load test.

### Fracture load testing

Following the storage period, fracture load testing was performed using a universal testing machine (Autograph AG-X; Shimadzu Corp., Kyoto, Japan), in accordance with the ISO 6872:2015 standard. The samples were securely mounted in the machine to ensure stable positioning during testing. The testing procedure involved the application of a compressive force to the occlusal surface of each endocrown, using a four mm diameter indenter that was selected to provide a broader and more standardized occlusal contact area at the central fossa, in line with previously published fracture load protocols (Badr et al., 2022; Findakly & Jasim, 2019; Mertsöz, Ongun & Ulusoy, 2023) Fig. 3).

The force was applied at a consistent crosshead speed of one mm/min following the guidelines specified in ISO 6872:2015 for ceramic materials testing parameters. This approach allowed controlled compressive loading until failure. The force was directed perpendicularly to the occlusal surface of the endocrowns, targeting the central fossa to simulate functional occlusal loading. The test continued until failure occurred, which was defined as the first sharp drop in the load–displacement curve, confirmed by visible crack formation, fracture, complete dislodgement of the endocrown from the tooth structure, and/or an audible fracture sound. The maximum force recorded immediately before this drop was recorded as the fracture load for each specimen. This value represented the peak force that the endocrown could withstand before structural failure occurred. After fracture testing, failure patterns were categorized according to whether damage was confined to the restoration or involved the tooth structure. The fractured specimens were then evaluated independently by two calibrated examiners under 5.0 × loupe magnification (ZEISS EyeMag Pro S, Carl Zeiss Meditec AG, Jena, Germany) and further descriptively classified as simple or complex/destructive failure patterns. Simple failures were defined as restoration-confined fractures or minor enamel cracks without substantial loss of tooth structure, or debonding/dislodgement of the restoration without involvement of the underlying tooth structure. These failures were considered potentially repairable. Complex/destructive failures were defined as failures involving extensive tooth structure damage, with fracture propagation through dentine and extension to the cementoenamel junction (CEJ) region, with or without root involvement. These failures were considered potentially non-repairable because of the extent of structural damage and the unfavorable fracture extension. Inter-examiner agreement was assessed using Cohen’s kappa, and disagreements, if any, were resolved by discussion until consensus was reached.

### Statistical analysis

Statistical analysis was performed using SPSS software version 26.0 (IBM Corp., Armonk, NY, USA). Data were initially assessed for normality using the Kolmogorov–Smirnov and Shapiro–Wilk tests. The fracture load data for both groups were normally distributed (*p* > 0.05), supporting the use of parametric statistical analysis. Descriptive statistics included mean, standard deviation, median, and 95% confidence interval. Intergroup comparison of fracture load was performed using an independent samples *t*-test. As Levene’s test indicated unequal variances, Welch’s *t*-test was applied. Failure pattern, classified as simple or complex/destructive, was compared between groups using Fisher’s exact test because of the small sample size and expected cell counts below 5. A *p*-value of less than 0.05 was considered statistically significant.

## Results

An independent samples *t*-test was performed to evaluate the difference in fracture load between the two groups. Levene’s test indicated unequal variances (*p* = 0.003); therefore, the Welch’s *t*-test was applied. The analysis revealed a highly significant difference between the ZR and VS groups (*t* = 8.257, *df* = 8.566, *p* < 0.001) (Table 2).

**Table 2:** Independent samples *t*-test comparing fracture load between the two different materials.

Statistic	Value
Levene’s Test for Equality of Variances (F, p)	11.714, *p* = 0.003
*t*-value	8.257
Degrees of freedom (Welch’s df)^*^	8.566
*p*-value (two-tailed)	<0.001
Mean difference (N)	2,220.27
Standard error of the difference (N)	268.89
95% Confidence interval of the difference (N)	1,607.27–2,833.26

**Notes.**

* Levene’s test was significant; therefore, equal variances not assumed (Welch’s *t*-test).

The results of this study demonstrated a significant difference in fracture load between the ZR and VS groups. The ZR group showed markedly higher values, with a mean fracture load of 3615.36 N (SD 792.74 N) and a 95% confidence interval (CI) of 3,006.00–4,224.71 N. The median value was 3,746.70 N, and fracture load ranged from 2,272.15 N to 4,605.24 N (range 2,333.09 N).

**Figure 4:**
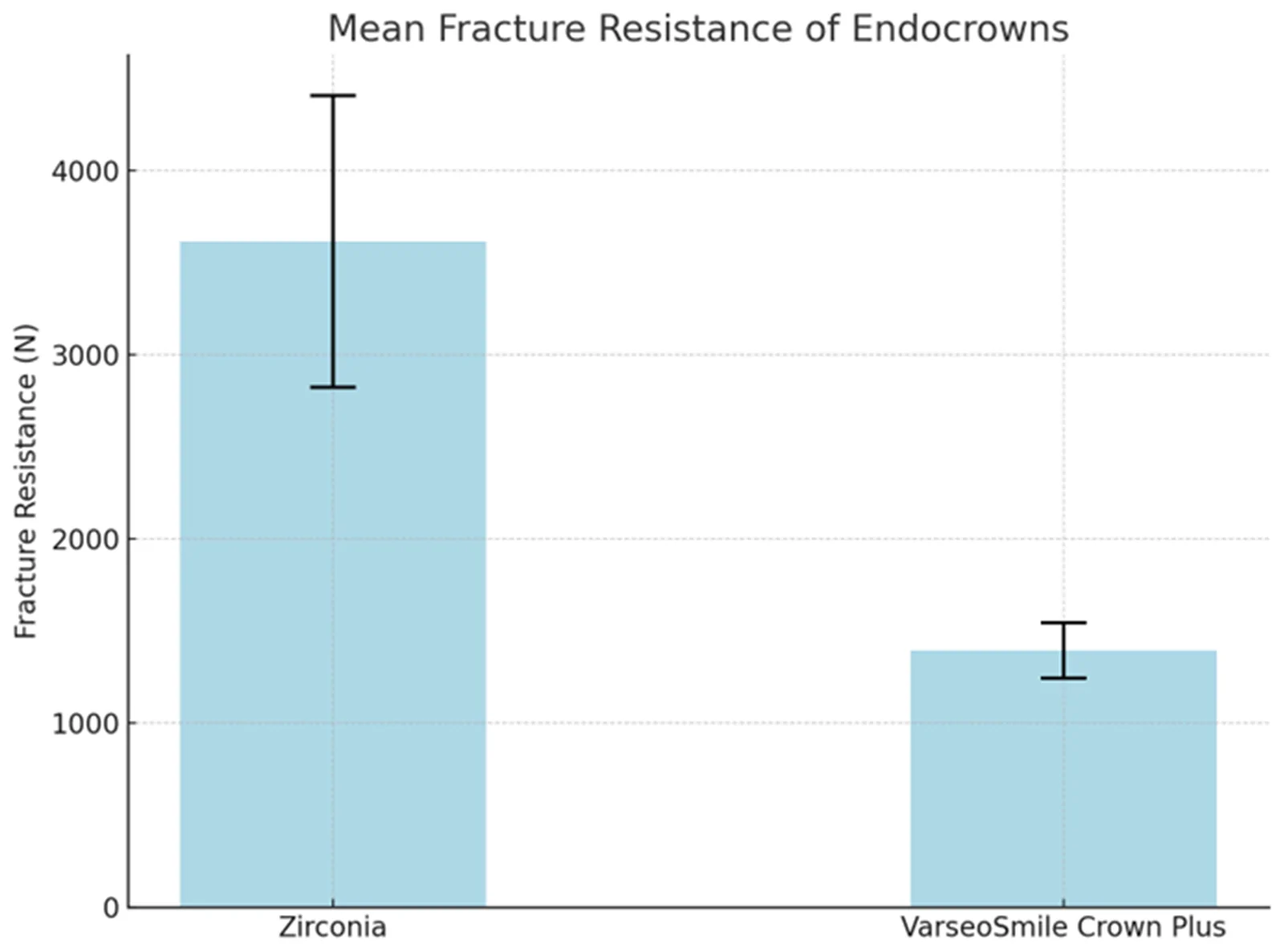
Comparison of mean fracture load (N) between ZR and VS groups, with standard deviations.

The VS group exhibited a considerably lower mean fracture load of 1,395.09 N (SD = 149.24 N). The 95% CI for the VS group was narrower, ranging from 1,280.37 N to 1,509.81 N, indicating less variability in the fracture load values. The median fracture load was 1,349.92 N, closely aligning with the mean, further supporting the symmetrical distribution of the data. The minimum and maximum fracture load values for the VS group were 1,213.06 N and 1,576.79 N, respectively, with a range of 363.73 N, reflecting a more consistent performance across samples compared with the ZR group (Table 3 and Fig. 4).

**Figure 5:**
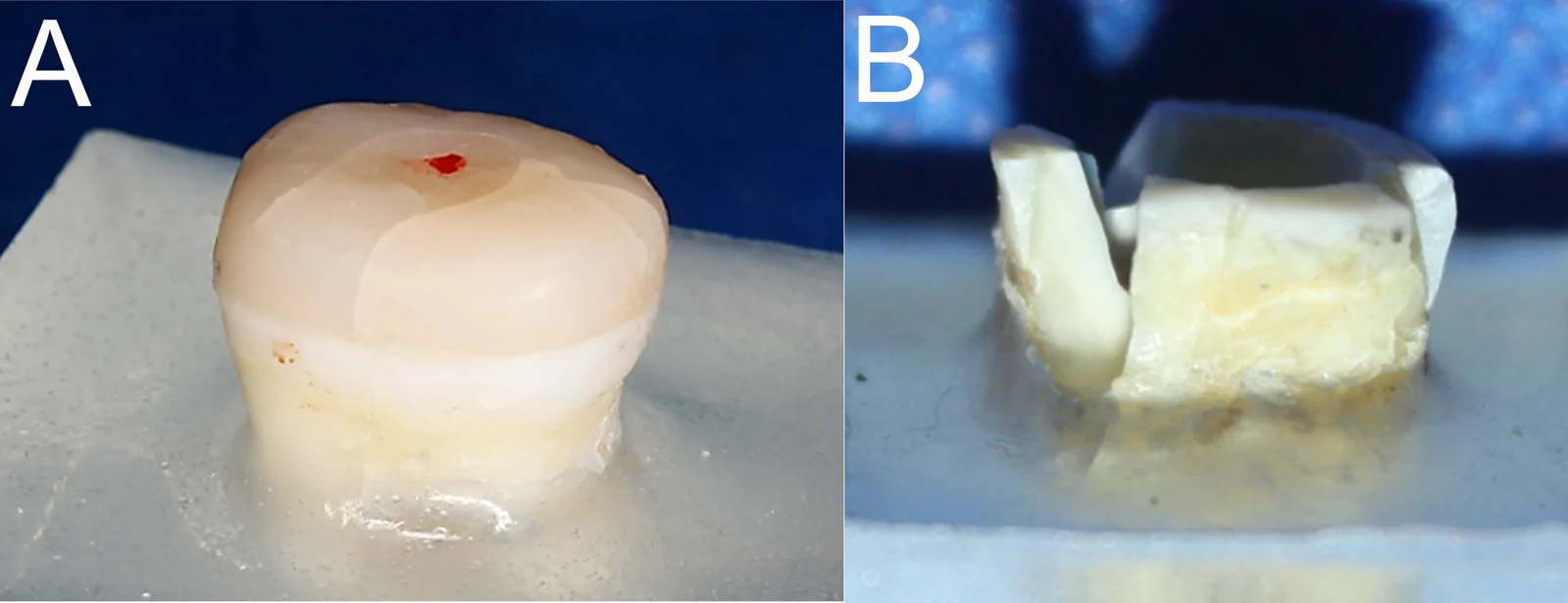
Representative fracture patterns of the samples. Representative fracture patterns. (A) 3D-printed resin (VS) showing a simple restoration-confined failure pattern with no involvement of the natural tooth structure; (B) ZR specimen illustrating a complex/destructive tooth-involving failure pattern.

Inter-examiner agreement for failure classification was high, with a Cohen’s kappa value of 0.89, indicating very high agreement between the two evaluators. The fracture behavior differed between groups (Fig. 5). In the ZR group, fractures involving the natural tooth structure were observed in 8/9 specimens (88.9%); of these, five specimens (55.6% of the total group) were classified as complex/destructive failures and were considered potentially non-repairable. In the VS group, restoration-confined failures occurred in 5/9 specimens (55.6%) and were considered potentially repairable, whereas tooth structure involvement was observed in 4/9 specimens (44.4%); all the tooth-involving failures in the VS group were simple enamel cracks. Fisher’s exact test showed a statistically significant difference in failure pattern distribution between the ZR and VS groups (*p* = 0.029) (Table 4).

**Table 3:** Descriptive statistics of fracture load values for zirconia and VarseoSmile Crown Plus endocrowns.

Material	Mean (N)	Standard deviation (N)	95% confidence interval (N)
Zirconia	3,615.36^a^	792.74	3,006.00 –4224.71
VarseoSmile Crown Plus	1,395.09^b^	149.24	1,280.37 –1509.81

**Notes.**

Different superscript letters indicate a statistically significant difference between groups based on Welch’s *t*-test (*p* < 0.05).

**Table 4:** Distribution of failure modes in zirconia and 3D-printed resin endocrowns.

**Failure mode**	**ZR, n (%)**	**VS, n (%)**	**Statistical comparison**
Restoration-confined fracture	1/9 (11.1%)	5/9 (55.6%)	
Enamel crack only	3/9 (33.3%)	4/9 (44.4%)	
Tooth-involving failures	8/9 (88.9%)	4/9 (44.4%)	
Complex/destructive failure	5/9 (55.6%)	0/9 (0.0%)	
Debonding/dislodgement	0/9 (0.0%)	0/9 (0.0%)	
Overall simple/potentially repairable failure	4/9 (44.4%)	9/9 (100.0%)	Fisher’s exact test, *p* = 0.029
Overall complex/potentially non-repairable failure	5/9 (55.6%)	0/9 (0.0%)	

**Notes.**

ZR: zirconia endocrowns; VS: 3D-printed resin endocrowns. Values are presented as frequency/total number and percentage. Fisher’s exact test was used to compare the overall distribution of simple/potentially repairable and complex/potentially non-repairable failure patterns between groups.

## Discussion

The results of this study provide valuable insights into the fracture load of endocrowns fabricated from two different materials: multilayer zirconia and the tested 3D-printed resin material. The findings reveal a significant difference in the fracture load between the two materials, with zirconia demonstrating a notably higher mean fracture load compared with the tested 3D-printed material. This difference highlights the inherent material properties that contribute to the mechanical performance of endocrown restorations.

A literature based endocrown preparation design was adopted in the present study rather than an arbitrary protocol. The selected two mm occlusal reduction and four mm intracoronal extension are consistent with preparation dimensions reported in experimental endocrown studies, supporting the clinical and methodological validity of the present design (Amjadi et al., 2023; Hayes et al., 2017; Mertsöz, Ongun & Ulusoy, 2023).

In terms of material selection, the present study also highlights the mechanical performance of the tested 3D-printed resin material, a ceramic filled definitive 3D-printed resin developed for single unit indirect restorations, including crowns, inlays, and onlays. According to manufacturer data, it exhibits a flexural strength of 116 to 150 MPa and a flexural modulus of approximately 4.09 GPa, indicating mechanical properties that are substantially lower than those of zirconia but still within the range reported for definitive printed restorative resins. Chemically, VS is a methacrylate-based, ceramic-filled hybrid resin. According to the manufacturer, it contains esterification products of ethoxylated bisphenol A dimethacrylate, silanised dental glass, methyl benzoylformate, and diphenyl(2,4,6-trimethylbenzoyl) phosphine oxide, with an inorganic filler content of 30–50 wt% and a particle size of approximately 0.7 µm (BEGO, 2025). Previous investigations have also reported encouraging findings regarding its clinical applicability. Al-Ramadan et al. found that VarseoSmile Crown Plus and CROWNTEC showed internal and marginal fit comparable with milled Vita Enamic, although Vita Enamic demonstrated superior trueness and precision in fabrication accuracy (Al-Ramadan et al., 2024). Published evidence further suggests that the mechanical performance of VS is variable and strongly influenced by testing and manufacturing conditions. Di Fiore et al. reported a mean flexural strength of 109.9 ± 15.8 MPa under dry conditions, which decreased to 94.2 ± 11.7 MPa after artificial ageing; in the same study, Vickers hardness decreased from 31.5 ± 0.6 to 29.6 ± 1.0, while elastic modulus decreased from 3.82 ± 0.2 GPa to 3.59 ± 0.2 GPa, confirming measurable degradation after ageing (Di Fiore et al., 2024). Similarly, Grzebieluch et al. (2021) reported flexural strength values of 119.85 ± 17.95 MPa and 143.39 ± 12.88 MPa for VS, with the difference attributed to specimen orientation during printing. More recent evidence has confirmed that the material is technique-sensitive, as print orientation significantly affects flexural strength and flexural modulus, with specimens printed at 90° generally performing better than those printed at 0° or 45°, while ageing in water or artificial saliva further reduces these properties (Mudhaffer et al., 2025). In addition, the post-washing protocol has been shown to influence flexural strength, elastic modulus, hardness, water sorption, and solubility, highlighting the importance of post-processing for long-term mechanical stability (Di Fiore et al., 2025). Optical properties have also been investigated, and Tokar et al. (2024) reported that VS showed the highest translucency among the tested materials, although it exhibited lower flexural strength, indicating potential limitations in high load-bearing applications. Taken together, these findings suggest that the tested 3D printed resin in this study can be considered a promising definitive 3D-printed restorative material, with acceptable fit, translucency, and digital workflow advantages; however, its mechanical reliability remains lower than that of zirconia and appears to be strongly influenced by ageing and manufacturing variables (Di Fiore et al., 2024; Di Fiore et al., 2025; Mudhaffer et al., 2025).

Previous *in vitro* studies have generally shown that zirconia endocrowns exhibit high fracture load, particularly in molars. Sahebi et al. (2022) reported that zirconia endocrowns had significantly higher fracture load than zirconium lithium silicate endocrowns after thermomechanical ageing, with mean values of 7,395.07 ± 1,947.42 N and 1,618.3 ± 585 N, respectively. Similarly, Amjadi et al. (2023) found that zirconia endocrowns in mandibular molars showed a mean fracture load of 5,374.68 ± 670.03 N, significantly higher than zirconia onlays (3,312.50 ± 804.01 N).

The superior fracture load shown by the ZR group, reflected in its higher mean value (3,615.36 N, SD = 792.74 N) and wider fracture load range (2,272.15 N to 4,605.24 N), can be attributed to its well-documented mechanical advantages, particularly its high flexural strength and fracture toughness. These properties contribute to zirconia’s capacity to endure substantial occlusal forces, supporting its use as a reliable material for posterior restorations (Huang et al., 2024). According to Huang et al. (2024) zirconia demonstrates superior mechanical characteristics, including high flexural strength and fracture toughness, which significantly enhance its resistance to crack propagation and mechanical failure. Similarly, Abad-Coronel et al. (2025) reported that monolithic zirconia exhibits excellent fracture load and reliability under occlusal stress, supporting its clinical effectiveness in high-load bearing posterior restorations.

Although the tested 3D-printed resin material demonstrated lower fracture load than zirconia, its mean fracture load (1,395.09 N) remained above the average maximum bite force commonly reported in the molar region, which is generally around 500 to 700 N in healthy adults (Bakke, 2006; Nitschke et al., 2025; Shoji et al., 2022). However, parafunctional activity such as bruxism may generate substantially higher occlusal loads, and this should be considered when selecting the material for high-stress clinical situations (Bakke, 2006; Nitschke et al., 2025; Shoji et al., 2022).

While its mechanical limitations may restrict its use in high-stress cases such as patients with bruxism, it remains a viable option in situations where rapid fabrication, aesthetic outcomes, and workflow efficiency are prioritized. The narrower confidence interval and lower variability within this group suggest that the tested 3D-printed resin showed more consistent performance across different samples, although it does not match the fracture load levels of zirconia. The statistically significant difference in fracture load between the two materials, as demonstrated by Welch’s *t*-test (*p* < 0.001), highlights the critical importance of material selection in endocrown restorations. While zirconia offers superior mechanical performance, the optimal choice of material should be informed by a comprehensive evaluation of the clinical case, balancing functional demands with aesthetic considerations and patient-specific factors. This underscores the need for an individualized, evidence-based approach to restorative material selection to ensure long-term clinical success (Abad-Coronel et al., 2025).

The findings of this study align with previous research that has highlighted zirconia’s exceptional mechanical properties, further reinforcing its role as a reliable material for durable restorations. However, the study also contributes to the growing body of evidence supporting the use of newer ceramic-filled resin materials in specific clinical contexts, particularly where advanced manufacturing techniques such as 3D-printing can offer practical benefits (Abad-Coronel et al., 2025; Graf et al., 2022; Huang et al., 2024).

Despite its higher fracture load, the ZR group showed a tendency toward more severe failure patterns, with a high proportion of fractures extending into the tooth structure and more than half classified as complex or destructive. From a clinical standpoint, such patterns may be less favorable because they are more likely to compromise the remaining tooth substrate and reduce the potential for conservative repair (El-Damanhoury, Haj-Ali & Platt, 2015; Huang et al., 2024; Zarone et al., 2019). In contrast, the VS group more frequently demonstrated restoration confined fractures and, when tooth involvement occurred, it was predominantly limited to simple enamel cracking. This pattern aligns with reports that resin based, or ceramic filled 3D-printed restorative materials exhibit comparatively greater compliance and may dissipate functional stresses more favorably, which can translate into less severe fracture presentations under static loading (Abad-Coronel et al., 2025; Tokar et al., 2024). This pattern suggests a potentially more repairable failure profile for the printed resin material under the present testing conditions (Fig. 5).

While this study offers valuable insights into the mechanical performance of the tested 3D-printed resin material, certain limitations should be noted. The relatively small sample size may limit the generalizability of the findings, and the study was not designed to provide a broader comparison across multiple restorative materials. As an *in vitro* investigation, the study does not account for clinical factors such as occlusal variability, parafunctional habits, the dynamic oral environment, or periodontal ligament simulation. Factors such as moisture, thermal fluctuations, cyclic loading, and post-processing conditions may influence material performance. In particular, the washing protocol and alcohol concentration used during post-processing of 3D-printed resin restorations should be considered as methodological factors that may affect their mechanical and physical properties. In addition, no additional airborne-particle abrasion, etching, or primer application was performed on the intaglio surface before cementation, as the primary outcome was fracture resistance under compressive loading rather than bond strength or cement retention. However, the absence of surface treatment may have influenced bonding behavior and fracturing outcomes and should be considered in future studies evaluating bonding durability and long-term performance. Previous research has shown that cyclic loading reduces fracture load in both milled and 3D-printed zirconia crowns, with lower load in 3D-printed versions (Refaie et al., 2023), whereas hydrothermal aging compromises the flexural strength of 3D-printed resins due to water uptake and surface degradation (Nam et al., 2021). In addition, failure analysis in the present study was based on visual examination under loupe magnification and descriptive classification only, without complementary fractographic analysis such as SEM; therefore, the precise origin and propagation pathway of fracture could not be determined. Further studies with larger sample sizes, ageing protocols, a wider range of restorative materials, and additional reliability analyses such as Weibull analysis are needed to better clarify fracture behavior and long-term clinical performance. In addition, the selected preparation design was used as a standardized experimental model and may not fully represent severely compromised or highly non-retentive clinical endocrown situations.

## Conclusion

In conclusion, zirconia endocrowns demonstrated higher fracture load than the tested 3D-printed resin material under the conditions of this *in vitro* study. However, the 3D-printed restorations showed a greater proportion of simple, potentially repairable failure patterns, as fractures were more frequently confined to the restoration rather than involving the tooth structure. These findings suggest that material selection should consider not only fracture load but also failure behavior and the clinical requirements of each case. Within the limitations of this study, the tested 3D-printed resin material showed encouraging *in vitro* performance, and it may be considered as alternative in selected situations. Further studies are needed to assess the long-term performance of these materials under ageing and functional loading conditions.

## Supplemental Information

10.7717/peerj.21602/supp-1 Supplemental Information 1 Raw fracture resistance values (N) by group Raw fracture resistance values (N) for both groups zirconia endocrowns and 3d-printed endocrowns

## Additional Information and Declarations

### Data Availability

The following information was supplied regarding data availability: The raw fracture resistance values are provided in the Supplemental File.
